# Managing burn wounds with SMARTPORE Technology polyurethane foam: two case reports

**DOI:** 10.1186/s13256-016-0918-3

**Published:** 2016-05-12

**Authors:** Farrah-Hani Imran, Rahamah Karim, Noor Hidayah Maat

**Affiliations:** 1 Head of Plastic & Reconstructive Surgery, Burns and Wound Care, Department of Surgery, Faculty of Medicine, Universiti Kebangsaan Malaysia Medical Centre (UKMMC), Kuala Lumpur, Malaysia; 2 Burns Unit, UKMMC, Kuala Lumpur, Malaysia

**Keywords:** Case report, Atraumatic dressing removal, Burn wounds, Exudate, Exudating wound, Wound dressing, SMARTPORE Technology polyurethane foam

## Abstract

**Background:**

Successful wound healing depends on various factors, including exudate control, prevention of microbial contaminants, and moisture balance. We report two cases of managing burn wounds with SMARTPORE Technology polyurethane foam dressing.

**Case presentation:**

In Case 1, a 2-year-old Asian girl presented with a delayed (11 days) wound on her right leg. She sustained a thermal injury from a hot iron that was left idle on the floor. Clinical inspection revealed an infected wound with overlying eschar that traversed her knee joint. As her parents refused surgical debridement under general anesthesia, hydrotherapy and wound dressing using SMARTPORE Technology Polyurethane foam were used. Despite the delay in presentation of this linear thermal pediatric burn injury that crossed the knee joint, the patient’s response to treatment and its outcome were highly encouraging. She was cooperative and tolerated each dressing change without the need of supplemental analgesia. Her wound was healed by 24 days post-admission. In Case 2, a 25-year-old Asian man presented with a mixed thickness thermal flame burn on his left leg. On examination, the injury was a mix of deep and superficial partial thickness burn, comprising approximately 3 % of his total body surface area. SMARTPORE Technology polyurethane foam was used on his wound; his response to the treatment was very encouraging as the dressing facilitated physiotherapy and mobility. The patient rated the pain during dressing change as 2 on a scale of 10 and his pain score remained the same in every subsequent change. His wound showed evidence of epithelialization by day 7 post-burn. There were no adverse events reported.

**Conclusions:**

Managing burn wounds with SMARTPORE Technology polyurethane foam resulted in reduced pain during dressing changes and the successful healing of partial and mixed thickness wounds. The use of SMARTPORE Technology polyurethane foam dressings showed encouraging results and requires further research as a desirable management option in burn wounds.

## Background

Wound healing is a natural process in the human body to restore injured tissue. It consists of four overlapping phases [1, 2]:

- Phase 1 – Hemostasis (clot formation by platelets)
- Phase 2 – Inflammation (debridement of injured tissue by inflammatory cells)
- Phase 3 – Proliferation (occurrence of epithelialization, fibroplasia, and angiogenesis, formation of granulation tissue, and initiation of wound contraction) and
- Phase 4 – Remodeling (restoration of the tensile strength of injured skin through collagen formation)

Wound healing is successful when these four phases occur in a proper sequence and timeframe [2]. In contrast, wound healing can be delayed under the influence of conditions such as desiccation, infection or presence of abnormal bacteria, maceration, necrosis, pressure, trauma, and edema [3].

Chronic wounds are commonly associated with copious exudates that can cause leakage, skin problems, electrolyte imbalance, infection, odor, and psychological problems. Conversely, minimal exudates can delay autolytic debridement, inhibit epithelialization, and cause pain on dressing removal. Therefore, maintaining moisture balance in wounds is essential in wound management [4]. Wound dressings have always been the first-line management option in wound care as they can control moisture balance in wounds [4]. There are many different types of dressings, but an optimal wound dressing should have the following characteristics [5, 6]:

- Able to control exudates without drying out the wound surfaces.
- Can act as a barrier to external contaminants.
- Allows atraumatic removal with no dressing left in the wounds.
- Provides moisture vapor permeability sufficient to prevent over-hydration of the wound and surrounding skin.
- Reduces pain during dressing change.

SMARTPORE Technology polyurethane foam is an example of foam dressing that has these characteristics. The purpose of this case report is to describe the clinical presentation, objective findings, and outcomes of burn wound management for one pediatric and one adult using SMARTPORE Technology polyurethane foam.

## Case presentation

### Case 1

A 2-year-old Asian girl presented to us 13 months ago with a delayed wound on her right leg. Eleven days prior to her arrival at our hospital she sustained a thermal injury from a hot iron that was left idle on the floor. Her parents did not recall any first aid measures; they applied topical oil on the burn area and expected that the wound would heal naturally. As the wound failed to progress, they decided to bring her to our Emergency Department for further assessment and management. Upon arrival, she was pyrexial but comfortable. Wound inspection revealed an infected wound with overlying eschar on her right lower limb that traversed her knee joint. The total body surface area (TBSA) affected was approximately 5 %. She was not on any medication and had no known drug allergies. Her hematological parameters were within the normal range. She was commenced on analgesia as necessary and antibiotics were administered intravenously based on wound culture and sensitivities.

**Fig. 1:**
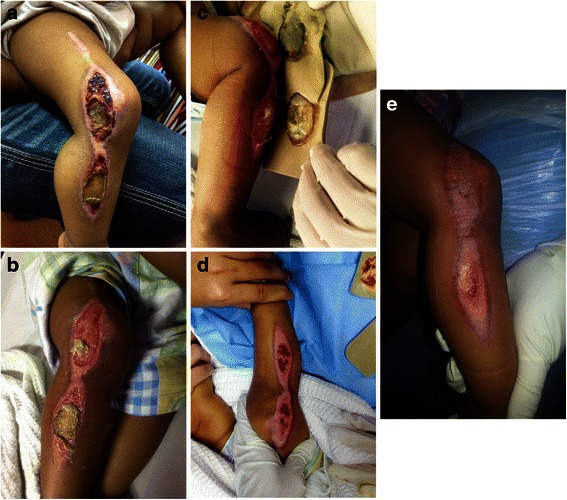
Sequential recovery from burn injury in a 2-year-old patient using SMARTPORE Technology polyurethane foam. **a** On admission, post-burn day 11. **b** Post-burn day 18. **c** Post-burn day 23. **d** Post-burn day 30. **e** Post-burn day 35. ©Plastic Surgery, Burns & Wound Care Unit, Universiti Kebangsaan Malaysia

She was admitted and seen by a multidisciplinary wound care team comprising a physiotherapist, dietitian, pediatrician, and plastic surgeon. Her parents refused surgical debridement under general anesthesia. As a compromise, hydrotherapy and wound dressing with advanced wound care were performed. Her fever resolved spontaneously by day 3 of admission. As the wound was gently dressed over the next few days, it became clear that the burn was a deep partial thickness burn. Escharectomy (removal of devitalized tissue and debris) was performed by serial wound dressings. Hydrocolloid gel was used as a primary dressing, while SMARTPORE Technology polyurethane foam was the secondary dressing in the first 5 days. Once the eschar was fully removed, SMARTPORE Technology polyurethane foam was continued every other day until the wound healed (Fig. 1). Two separate pieces of SMARTPORE Technology polyurethane foam were used to facilitate full range-of-movement at her knee joint for physiotherapy. Hydrotherapy and cleansing of her wound at each dressing change were performed with antiseptic solution and saline. Dressing change was painless as the SMARTPORE Technology polyurethane foam did not adhere to the wound surface. It also demonstrated good absorptive qualities. This facilitated the serial removal of the eschar and slough via atraumatic mechanical debridement. Despite the delay in presentation of this linear thermal pediatric burn injury that crossed the knee joint, the patient’s response to treatment and its outcome were highly encouraging. She was cooperative and tolerated each dressing change without the need of supplemental analgesia. This was coupled with positive feedback from the parents and ward nurses, as the use of SMARTPORE Technology polyurethane foam eased the dressing changes. Her wound was healed by 24 days post-admission. Her parents were advised on scar management, which included a customized pressure garment and continued physiotherapy.

### Case 2

**Fig. 2:**
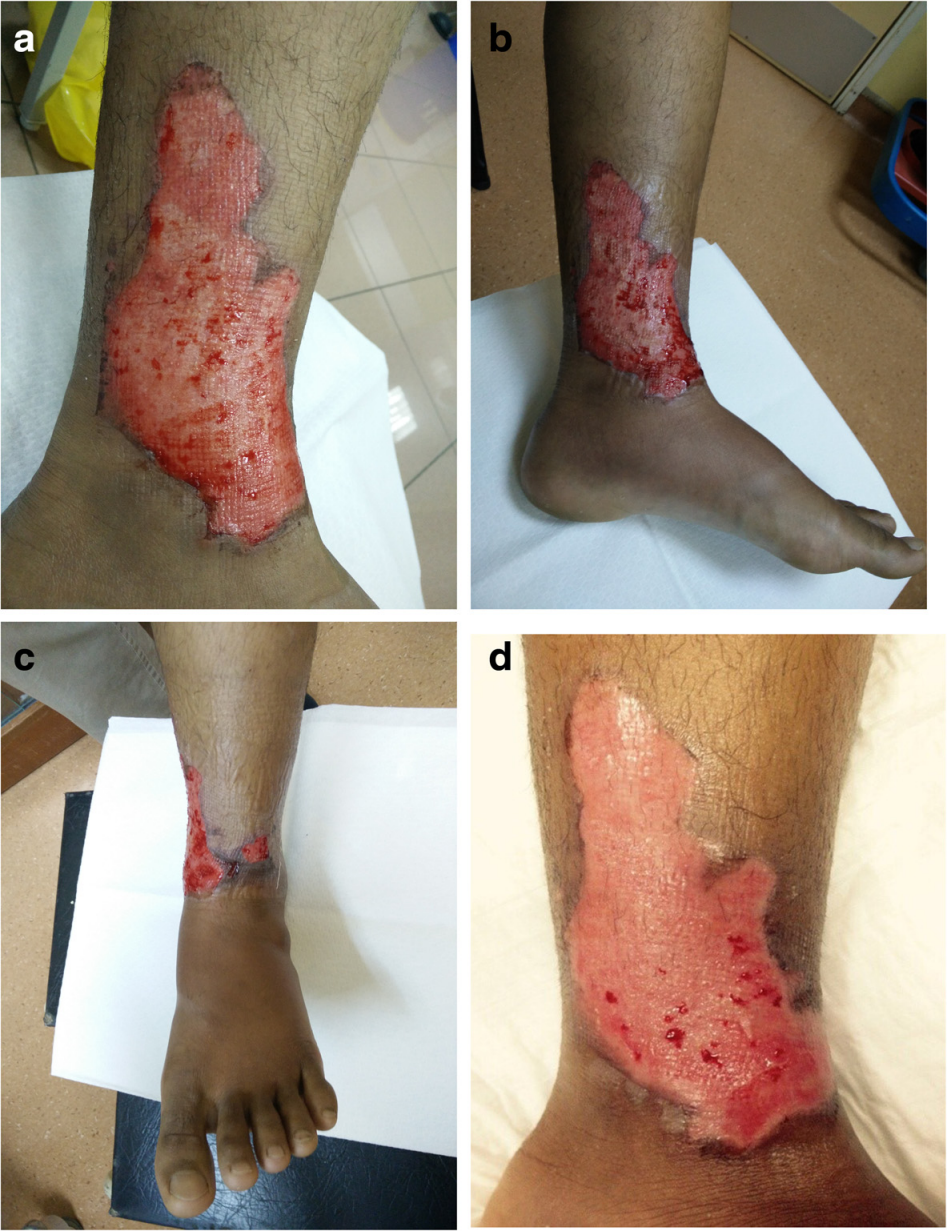
Sequential recovery from burn injury in an adult patient using SMARTPORE Technology polyurethane foam. **a** Post-burn day 1. **b** Post-burn day 3. **c** Post-burn day 5. **d** Post-burn day 7. © Plastic Surgery, Burns & Wound Care Unit, Universiti Kebangsaan Malaysia

A 25-year-old Asian man presented with a mixed thickness thermal flame burn on his left leg 12 months ago. Prior to his arrival at the hospital, he applied toothpaste to the wound as a first aid measure. On examination, the injury was a mix of deep and superficial partial thickness burns with affected TBSA of approximately 3 %. The wound was clean and surrounded by healthy tissues. He was not on any medication and had no known drug allergies. All hematological investigations revealed normal findings with no clinical signs of infection. He was admitted to our Burn Unit for hydrotherapy and advanced multidisciplinary wound care. SMARTPORE Technology polyurethane foam was used on his wound and his response to the treatment was very encouraging. The only challenge was in educating him on the aim of multidisciplinary burn wound care and his role in burn wound management, that is, to be compliant with physiotherapy and to elevate his affected lower limb whenever possible. However, it was clear that the use of SMARTPORE Technology polyurethane foam facilitated physiotherapy and mobility. He rated his pain during dressing change as 2 on a scale of 10, and his pain score remained the same in every subsequent change. His wound showed evidence of epithelialization by day 7 post-burn (Fig. 2). There were no adverse events reported.

## Discussion

SMARTPORE Technology polyurethane foam is designed for the hydration and management of partial and full thickness wounds with exudates [7]. It is made up of three layers: a protection layer, an absorption layer, and a wound contact layer. The wound contact layer (inner layer) consists of uniform micropores that helps prevent new epithelial cells from growing into the dressing material. This keeps the skin from sticking to the dressing surface as it heals, leading to reduced pain during dressing change. The high absorption and retention capacity of the absorption layer (middle layer), on the other hand, allows maintenance of a proper moist condition and shortens wound healing time, as well as preventing leakage of exudates. The protection layer is the outermost layer, which functions to prevent external contamination and bacterial invasion [8].

Apart from wound dressings, multidisciplinary care also plays an important role in holistic assessment and treatment of patients with wounds. Apart from assessing and treating wounds, wound care also entails management of factors that could influence wound healing (for example, underlying disease, malnutrition, or possible infection) [9]. Notably, a single wound may benefit from different types of dressing as it progresses through the stages of wound healing. Thus, it is of paramount importance for the wound care team to be well versed with the spectrum of advanced dressings available, from a clinical efficacy and cost-benefit analysis point of view.

In the case of our patients, combining SMARTPORE Technology polyurethane foam as wound dressing with multidisciplinary care and current evidence-based practice resulted in good treatment outcomes. Limitations include planar wound-dressing interface and minimal range-of-movement restriction as the foam must be secured with a secondary dressing to keep it in place. Otherwise, the SMARTPORE Technology polyurethane foam has performed well in managing our patients’ wounds and its efficacy exceeded our expectations. Both patients were comfortable with the dressing. The patients reported minimal pain upon removal, facilitating a more pleasant experience during dressing changes, for both the patients and the attending nurses. It also showed highly satisfactory fluid absorption and exudate retention capacity. Nonetheless, it is important to note that wound management is commonly compromised by various co-morbidities, including the presence of infection, as well as vascular and systemic disease. Further comparison studies over a larger population would be required to further assess and definitively evaluate its efficacy in burn wound healing.

## Conclusions

Here, we presented two cases of burn wound management with SMARTPORE Technology polyurethane foam. The use of SMARTPORE Technology polyurethane foam dressings showed encouraging results and requires further research as a desirable management option in burn wounds.

## Consent

Written informed consent was obtained from the patient’s legal guardian for publication of the first case report and any accompanying images. A copy of the written consent is available for review by the Editor-in-Chief of this journal.

Written informed consent was obtained from the patient for publication of the second case report and any accompanying images. A copy of the written consent is available for review by the Editor-in-Chief of this journal.
